# Whole Exome Sequencing Identifies Three Novel Mutations in the *ASPM* Gene From Saudi Families Leading to Primary Microcephaly

**DOI:** 10.3389/fped.2020.627122

**Published:** 2021-02-11

**Authors:** Muhammad Imran Naseer, Angham Abdulrahman Abdulkareem, Osama Yousef Muthaffar, Sameera Sogaty, Hiba Alkhatabi, Sarah Almaghrabi, Adeel G. Chaudhary

**Affiliations:** ^1^Center of Excellence in Genomic Medicine Research, King Abdulaziz University, Jeddah, Saudi Arabia; ^2^Department of Medical Laboratory Technology, Faculty of Applied Medical Sciences, King Abdulaziz University, Jeddah, Saudi Arabia; ^3^Department of Biochemistry, Faculty of Science, King Abdulaziz University, Jeddah, Saudi Arabia; ^4^Department of Pediatrics, King Abdulaziz University, Jeddah, Saudi Arabia; ^5^Department of Medical Genetics, King Fahed General Hospital, Jeddah, Saudi Arabia; ^6^College of Applied Medical Sciences, King Abdulaziz University, Jeddah, Saudi Arabia; ^7^Center for Innovation in Personalized Medicine, King Abdulaziz University, Jeddah, Saudi Arabia

**Keywords:** *ASPM*, primary microcephaly, whole exom sequencing, Saudi population, MCPH

## Abstract

Autosomal recessive primary microcephaly (MCPH) is a neurodevelopmental defect that is characterized by reduced head circumference at birth along with non-progressive intellectual disability. Till date, 25 genes related to MCPH have been reported so far in humans. The *ASPM* (abnormal spindle-like, microcephaly-associated) gene is among the most frequently mutated MCPH gene. We studied three different families having primary microcephaly from different regions of Saudi Arabia. Whole exome sequencing (WES) and Sanger sequencing were done to identify the genetic defect. Collectively, three novel variants were identified in the *ASPM* gene from three different primary microcephaly families. Family 1, showed a deletion mutation leading to a frameshift mutation c.1003del. (p.Val335^*^) in exon 3 of the *ASPM* gene and family 2, also showed deletion mutation leading to frameshift mutation c.1047del (p.Gln349Hisfs^*^18), while in family 3, we identified a missense mutation c.5623A>G leading to a change in protein (p.Lys1875Glu) in exon 18 of the *ASPM* gene underlying the disorder. The identified respective mutations were ruled out in 100 healthy control samples. In conclusion, we found three novel mutations in the *ASPM* gene in Saudi families that will help to establish a disease database for specified mutations in Saudi population and will further help to identify strategies to tackle primary microcephaly in the kingdom.

## Introduction

Primary microcephaly (MCPH, OMIM 251200) is a congenital genetic neurodevelopmental disorder characterized by a small brain size along with mild to moderate intellectual disability. The head circumference (HC) is reduced from −4 SD (standard deviation) to −2 SD at the time of birth below the age and sex means as compared with the normal HC (1–3). In primary microcephaly, most of the patients have normal height, weight, and physical appearance as well as chromosomal banding pattern and brain scan result. In some of the cases, it is also observed that some patients show abnormal chromosomal analysis and short stature with mild to severe intellectual disability (1, 4). Studies on brain growth also revealed that primary microcephaly is due to the abnormal developmental process and not because of the degeneration or regression of the neuronal tissues (5, 6).

The occurrence of primary microcephaly is much more common in populations (ranging between 1.3 and 150 per 100,000 births) where the rate of consanguineous marriages is high such as in the Middle East and Asia, and it is much lower in Caucasians where consanguinity is lower (7). In the OMIM gene database, 25 genes associated with primary microcephaly have been reported so far.

MCPH1 is the most common type of primary microcephaly that occurs due to the causative variant of the microcephalin gene (known as MCPH1; 607117) located at chromosome 8p23. MCPH2 (604317) is due to any change in the *WDR62* gene (613583) that is positioned on 19q13 chromosome; MCPH3 (604804) occurs due to any mutation in the *CDK5RAP2* gene (608201) on 9q33; MCPH4 (604321) condition occurs due to any change in the *CASC5* gene (609173) located on 15q14 chromosome; MCPH5 (608716) is the most common condition of primary microcephaly that appears due to any change in the *ASPM* gene (605481) located on 1q31; MCPH6 (608393) is due to mutation in the *CENPJ* gene (609279) at 13q12 position; MCPH7 (612703) occurs due to any variation in the *STIL* gene (181590) on chromosome 1p33; MCPH8 (614673) is due to mutation in the *CEP135* gene (611423) on 4q12 chromosome; MCPH9 (614852) is due to mutation in the *CEP152* gene (613529) located on 15q21 chromosome; MCPH10 (615095) is due to any variation in the *ZNF335* (610827) gene on 20q13 chromosome; MCPH11 (615414) is due to mutation in the PHC1 *gene* (602978) on 12p13; MCPH12 (616080) condition is due to *CDK6* gene mutation (603368) on chromosome 7q21; MCPH13 (616051) condition leads to mutation in the *CENPE* (117143) gene on chromosome 4q24; MCPH14 (616402) is caused by any mutation in the *SASS6* gene (609321) on chromosome 1p21; and MCPH15 (616486) occurs due to any variation in the *MFSD2A* gene (614397) located on chromosome 1p34. In MCPH16 (616681) primary microcephaly, any mutation in the *ANKLE2* gene (616062) located on 12q24 chromosome leads to the disease; MCPH17 (617090) is due to variation in the *CIT* gene (605629) on chromosome 12q24; MCPH18 (617520) is due to any mutation in the *WDFY3* gene (617485) on 4q21; MCPH19 (617800) is linked to any variation in the *COPB2* gene (606990) on chromosome 3q23; MCPH20 (617914) occurs after any mutation in the *KIF14* gene (611279) located on 1q31; MCPH21 (617983) is due to mutation in the *NCAPD2* gene (615638) on 12p13; MCPH22 (617984) is due to any variation in the *NCAPD3* gene (609276) on 11q25; MCPH23 (617985) is any mutation in the *NCAPH* gene (602332) on 2q11 causing microcephaly; MCPH24 (618179) is linked to mutation in the *NUP37* gene (609264) on 12q23; and recently, MCPH25 (618351), linked to the primary microcephaly gene, is due to any mutation in the *MAP11* gene (618350) on 7q22 chromosome.

The *ASPM* gene (MIM# 605481) is positioned on chromosome 1q31.3, with a total number of 62,567 bp, along with 3,477 amino acids and a total of 28 exons (8, 9). During mitosis, the *ASPM* is present in spindle poles and centrosomes. The N-terminus of the *ASPM* gene domain includes 81 IQ (isoleucine and glutamine) along with the homology domain of calponin (10–12) and a C-terminus deprived of any domain ~220 kDa (13). The *ASPM* gene plays a vital role in the division of neural progenitor cells controlling cycling, by helping symmetric proliferative divisions as well as asymmetric neurogenic divisions (14). Animal model studies showed that knockdown of the *Aspm* gene leads to a decrease in cortical area and microcephaly as observed in humans (15, 16). The process through which the *Aspm* gene leads to microcephaly in mice is due to increasing the cell cycle duration in neural progenitors causing premature enervation of the neural progenitor pool and subsequently leading to a decrease in the upper layer of neuron production and an increase in the production at the lower end of the cortical area (15). Recently, we have reported novel mutations in genes such as *SATMBP* (17), *MCPH1* (18); (19), *WDR62* (20); (21), *PGAP2* (22), and *NT5C2* (23) related to microcephaly families. However, the mechanisms that explain the cause of microcephaly in humans are still unidentified.

Many of the *ASPM* mutations causing primary microcephaly have been reported so far. In the present study, we identified three families having novel variants in the *ASPM* gene that will be helpful for geneticists and clinicians to establish reliable diagnostic strategies for primary microcephaly families in Saudi population.

## Materials and Methods

### Sample Collection

We recruited three Saudi families with a clear phenotype of primary microcephaly. Pedigree was carefully constructed by interviewing the families. Peripheral blood samples of the affected and normal members of the families and from 100 healthy unrelated control were collected in EDTA tubes. Genomic DNA was extracted through QIAamp genomic DNA extraction or similar kits according to the manufacturers' protocols (https://www.qiagen.com/pk/products/top-sellers/qiaamp-dna-minikit/#orderinginformation). Genomic DNA quantification was performed with a Nanodrop spectrophotometer (https://www.thermofisher.com/order/catalog/product/ND-LITE-PR) and visualized with SYBR Safe (Thermo Fisher, USA) dye *via* running on 1% agarose horizontal gel electrophoresis apparatus. Informed consent from all the participants was obtained prior to the study. This study was permitted by the local ethical committee of King Abdulaziz University and followed all the guidelines according to the Declaration of Helsinki of 2013.

### Magnetic Resonance Imaging

Different pulse sequences in different imaging positions conducted utilizing MAGNETOM Symphony, A Tim System 1.5 T eco machine (Siemens) with a superconductive magnet was used for MRI analysis of the patients.

### Karyotype Analysis

Peripheral blood samples were used for karyotype analysis. Cell cultures were subjected to multiple steps including harvesting, preparation of the slide, staining, and microscopic analysis before obtaining the complete image of the chromosomes. Two lymphocyte cultures per patient were initiated in sterile T-25 flasks by adding 0.5–0.7 ml blood and 0.2 ml phytohemagglutinin solution to 10 ml of complete media consisting of 50 ml RPMI 1640, 10 ml fetal bovine serum (FBS), 0.5 ml penicillin/streptomycin solution, and 0.5 ml l-glutamine, and the cultures were incubated at 37°C for 72 h. After incubation for 48 h, 0.1 ml 5-fluoro-2-deoxyuridine (FUDR) solution was added into the thymidine culture flask and the contents were mixed gently. Following the incubation period, 0.2 ml of thymidine was also added and the contents were mixed and reincubated at 37°C. Three hours before cell harvest, 0.1 ml of ethidium bromide solution was added to the thymidine culture flask. Then, 0.65 ml of colcemid (10 μg/ml) was added to the regular flask and thymidine flask 10 and 15 min before cell harvest, respectively. After 25 min, the cultures were terminated and the contents of the two culture flasks were transferred into 15 ml centrifuge tube and centrifuged at 1,500 rpm for 10 min. Cells were fixed by adding 1 ml freshly made fixative. The fixed cell suspension was centrifuged at 1,500 rpm for 10 min, and the supernatant was removed, leaving ~1 ml, depending on the size of the pellet. The cells were resuspended using a narrow glass pipette. One to two drops of the cell suspension were dropped onto each marked slide from a height over vapor of hot water. Slides were left to dry at room temperature overnight. The spreads' quality and the mitotic index of the slide were checked using an Olympus phase contrast microscope (Model No. BX51TF). For G-banding and staining, four Coplin jars were prepared and each contained the following solution: Coplin jar 1 (48 ml of isotonic buffer + 2 ml of trypsin solution); Coplin jar 2 (50 ml isotonic buffer); Coplin jar 3 (50 ml Gurr buffer pH 6.8); and Coplin jar 4 (15 ml of refiltered Leishman stain + 45 ml Gurr buffer solution pH 6.8). As for chromosome trypsin treatment, the slides were dipped into Coplin jar 1 for 1 min. Then, the slides were soaked for 3 s in Coplin jars 2 and 3, respectively. The slides were stained with fresh Leishman stain for 2–3 min in Coplin jar 4. Immediately, the slides were rinsed in running water and air dried and observed using a Nikon microscope for the presence of optimal chromosome bands. The position of the metaphase spreads upon finding was captured by using the Cytovision tools, and the chromosomes of captured metaphases were separated, arranged in pairs (the pairs were compared band by band), and checked for any abnormality.

### Whole Exome Sequencing

To find out the pathogenic mutation that may be the cause of primary microcephaly, whole exome sequencing was performed for the affected members of the three families. The samples for sequencing were processed according to the guide from Agilent SureSelect Target Enrichment Kit (SureSelect_v6 Agilent USA), while whole exome sequencing was done using the Illumina HiSeq 2000/2500. The variants were filtered based on frequency, protein effect, quality, pathogenicity, genomic position, and relations with the disease phenotype. The variants or single nucleotide polymorphisms and short indel candidates are detected at nucleotide resolution at this stage. SNPs identified are compared to the 1000 Genomes (https://www.internationalgenome.org/), SnpEff (https://pcingola.github.io/SnpEff/), and genomAD databases (https://gnomad.broadinstitute.org/). We utilized various bioinformatics tools to find the causative variant for primary microcephaly such as the Laser gene Genomic Suite v. 12 (DNASTAR, Madison, WI, USA). Further, we used ArrayStar v. 12 (Rockville, MD, USA) to label variant alleles based on dbSNP142. The sequences obtained in FASTQ were further mapped to human reference genome which is termed as “alignment.” This alignment is performed through the Burrows-Wheeler arrangement tool (http://bio-bwa.sourceforge.net/bwa.shtml), and this alignment was done with human reference genome hg19 (http://hgdownload.cse.ucsc.edu/goldenPath). FASTQ raw data files are here converted to BAM files format. The BAM files obtained in the previous step were annotated with the Genome Analysis Toolkit (http://www.broadinstitute.org/gatk). It is an excellent and successful tool for variant discovery in next-generation sequencing data.

### Sanger Sequencing

Selected variants were confirmed through Sanger sequencing (SS) so that in the exome they have not been picked up as false positive. In SS, the first primers were selected through the Primer3Plus online software, and then the target DNA are PCR amplified, purified through PCR purification kits, and then Sanger sequenced through ABI genetic analyzers using the BigDye Terminator V3.1 Cycle Sequencing kit. For the mutation c.1003del, we used the forward primer sequence: 5′ CCGTAAATGTTAATGGCCAAAGA 3′, and the reverse primer sequence: 5′ ACATGTTTGCTGAGATGTACACA 3′; for the mutation c.1047del, we used the same forward primer sequence: 5′ CCGTAAATGTTAATGGCCAAAGA 3′, and the reverse primer sequence: 5′ CAGGAATACGTGGCGAAACT 3′; and for the mutation c.5623A>G, we used the forward primer sequence: 5′ ACGCCAGCTAATCAAACAACA 3′, and the reverse primer sequence: 5′ CTTCCTTCCTGCAGTCCATG 3′. These technologies provide sequence reads of 400–700 nucleotides, and thus, the target variants are easily confirmed through electropherograms obtained from the ABI genetic analyzer.

## Results

### Clinical Reports of the Patients

#### Family 1

**Figure 1:**
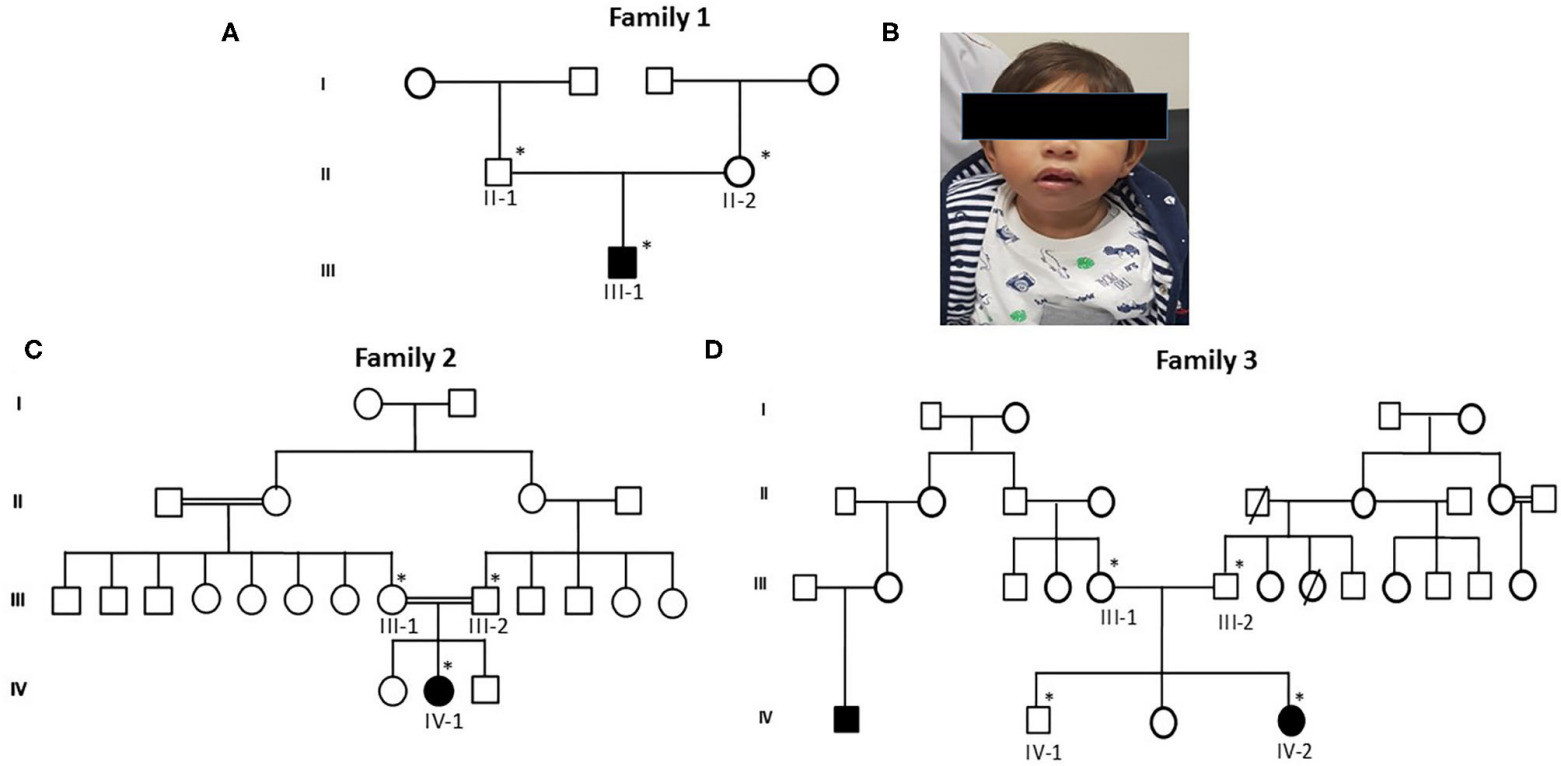
Pedigrees of primary microcephaly families **(A,C,D)** showing autosomal recessive mode of disease segregation. The open circles and squares showed unaffected females and males. Black filled circles and squares showing the affected members of the families. Double lines between circles and squares represent consanguineous marriage. The asterisk (*) sign represents the family members available for the study. **(B)** Patients' facial characteristics represented in photograph showing reduced head circumference and excluded facial dysmorphism.

Proband III-1, a boy, was 10 months old at the time of blood sample collection. He was the first baby of a non-consanguineous Saudi family as shown in Figure 1A. This was the first case in the family history. He has microcephaly of −4 SD measured as occipitofrontal head circumference along with mild to moderate intellectual disability. Furthermore, the typical feature of primary microcephaly with reduced head circumference but excluded facial dismorphism is shown in Figure 1B. He had no disorder of growth and no abnormal neurological findings and had normal chromosomes, and no metabolic as well as no neurological problem was observed. A karyotype study based on 15 metaphases analysis resulted as 46, XY normal male without any chromosomal changes. Brain MRI resulted in microcephaly with an abnormal triangular appearance of the frontal skull bones. There was preservation of the gray–white matter differentiation with no acute infraction, hemorrhage, hydrocephalus, brain herniation syndrome, or space-occupying lesion. The orbit, paranasal sinuses, and bones are unremarkable. The MRI report showed microcephaly with an abnormal triangular appearance of the frontal skull bones mostly related to premature closure of the metopic suture. There was no acute brain insult, mass effect, hydrocephalus, or space-occupying lesion. He was intellectually disabled. He has a typical feature of primary microcephaly like a sloppy forehead but with no other abnormal neurological finding, such as seizures, spasticity, or progressive cognitive failure.

#### Family 2

The second patient, proband IV-1, is a 5-year-old girl from a consanguineous family. Detailed family pedigree was drawn as shown in Figure 1C. Her head circumference measured 41 cm (−5 SD below the same age and sex). A karyotype study based on 15 metaphases analysis resulted as 46, XX normal female. She was mentally retarded by birth with clear features of primary microcephaly. Her facial features are like others previously reported with primary microcephaly such as a sloppy forehead and small boat-shaped dolichocephalic skull, the coronal sutures are relatively obliterated with no signs of increased ICT, etc. Her intelligence quotient (IQ) level was low. She was also not able to speak properly and not able to express her feelings as well. She had no other abnormal neurological findings, such as seizures, spasticity, or progressive cognitive debility. The MRI results showed severe hypogenesis of the cerebral hemispheres and cerebellum markedly affecting the occipital lobes. There was also severe hypogenesis of the corpus callosum area of the brain. The brain stem appears more or less normal in size and signal intensity. Finally, there was global brain hypogenesis markedly affecting the occipital lobes possibly due to intrauterine ischemic insult.

#### Family 3

Proband IV-2 is a 1-year-old girl at the time of sampling having clear features of primary microcephaly such as markedly bulging midface, small ears, floppy head, and curved nose. The detailed family history was taken and the pedigree was drawn as shown in Figure 1D. A karyotype study based on 15 metaphases analysis resulted as 46,XX normal female without any chromosomal changes. Her head size was below 3–4 standard deviations that is a clear indication of primary microcephaly. She was the third offspring delivered by non-consanguineous parents. At the age of 3, she was mentally retarded with very low IQ level, with no seizures but having behavioral abnormalities. The brother of the affected girl had borderline/normal intelligence, clumsiness, and episodic seizures, while her other sister had a normal IQ and no seizures and no behavioral abnormalities. The MRI report showed microcephaly with an abnormal triangular appearance of the frontal skull bones mostly related to premature closure of the metopic suture.

### Whole Exome Sequencing Analysis

For family 1, WES was done for proband III-1 and the resulting FASTQ files were converted to BAM and then the BAM files were converted to variant call format (vcf) file, obtaining 103,830 variants. These variants were utilized for the identification of mutation that may lead to the disease based on novel/rare (MAF + 0.01%) frequency, functionality (predicted to be damaging by PolyPhen/SIFT), homozygous/heterozygous state, genomic position, pathogenicity, protein effect, and earlier associations with the disease-related phenotype. We applied various filters and bioinformatics tools. The results revealed a homozygous frameshift mutation in the form of 1-bp deletion c.1003del. (p.Val335^*^) in exon 3 of the *ASPM* gene. The variant was not found in the large reference population cohort of the Genome Aggregation Database (gnomeAD, *n* > 120,000 exomes and >150,000 genomes). This variant generates a frameshift leading to a premature stop codon at position 335 in a new reading frame. It is predicted to be the basis for the loss of normal protein truncation (334 out of 3,477 aa) or nonsense-mediated mRNA decay. To best of our knowledge, the variant has not been reported in any medical literature or in a disease-related variation database like the Human Gene Mutation Database (HGMD) or ClinVar. However, an adjacent variant leading to a similar frameshift c.1002del, p. (Val335^*^) has been reported in a homozygous state in one family (24). Further, the WES for proband IV-1 from family 2 was done and the obtained vcf file showed 111,230 variants. The data analysis was done after applying various filters and bioinformatics tools (as explained above) and showed homozygous deletion variant in c.1047del G, p.Gln349HisfsTer18 in the *ASPM* gene in the affected child. Literature survey of this variants showed that the variant has not been reported in the literature or presented in disease-related variation database such as the HGMD or ClinVar. Moreover, the WES data for proband IV-2 from family 3 was also done and the resulting vcf files produced 96,301 variants. We did not find any homozygous mutation or heterozygous mutation in the family, but after applying various tools, the results showed a missense mutation c.5623A>G leading to a change in protein p.Lys1875Glu in exon 18 of the *ASPM* gene related to primary microcephaly.

### Sanger Sequencing

**Figure 2:**
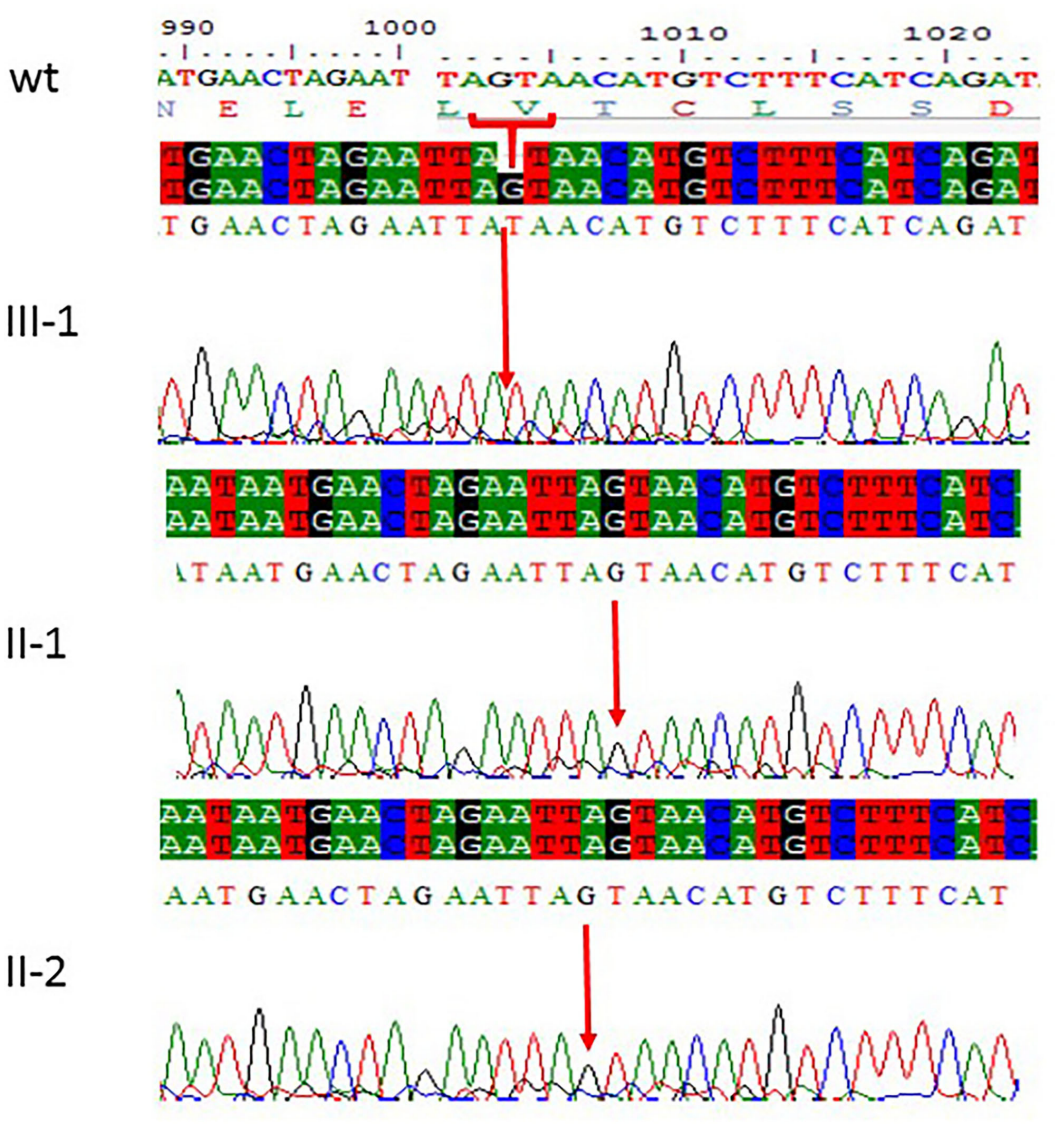
Sanger sequencing chromatograms showing c.1003del. (p.Val335*) in exon 3 of *ASPM* gene mutation in family 1. Proband III-1 showed deletion of “G” bp as compared with the wild-type control, while both parents II-1 and II-2 are homozygous at this position. The upper panel shows the sequencing of the affected member, whereas the lower panel shows the parents who are carrier individuals; the arrow is pointing at the site of mutation.

**Figure 3:**
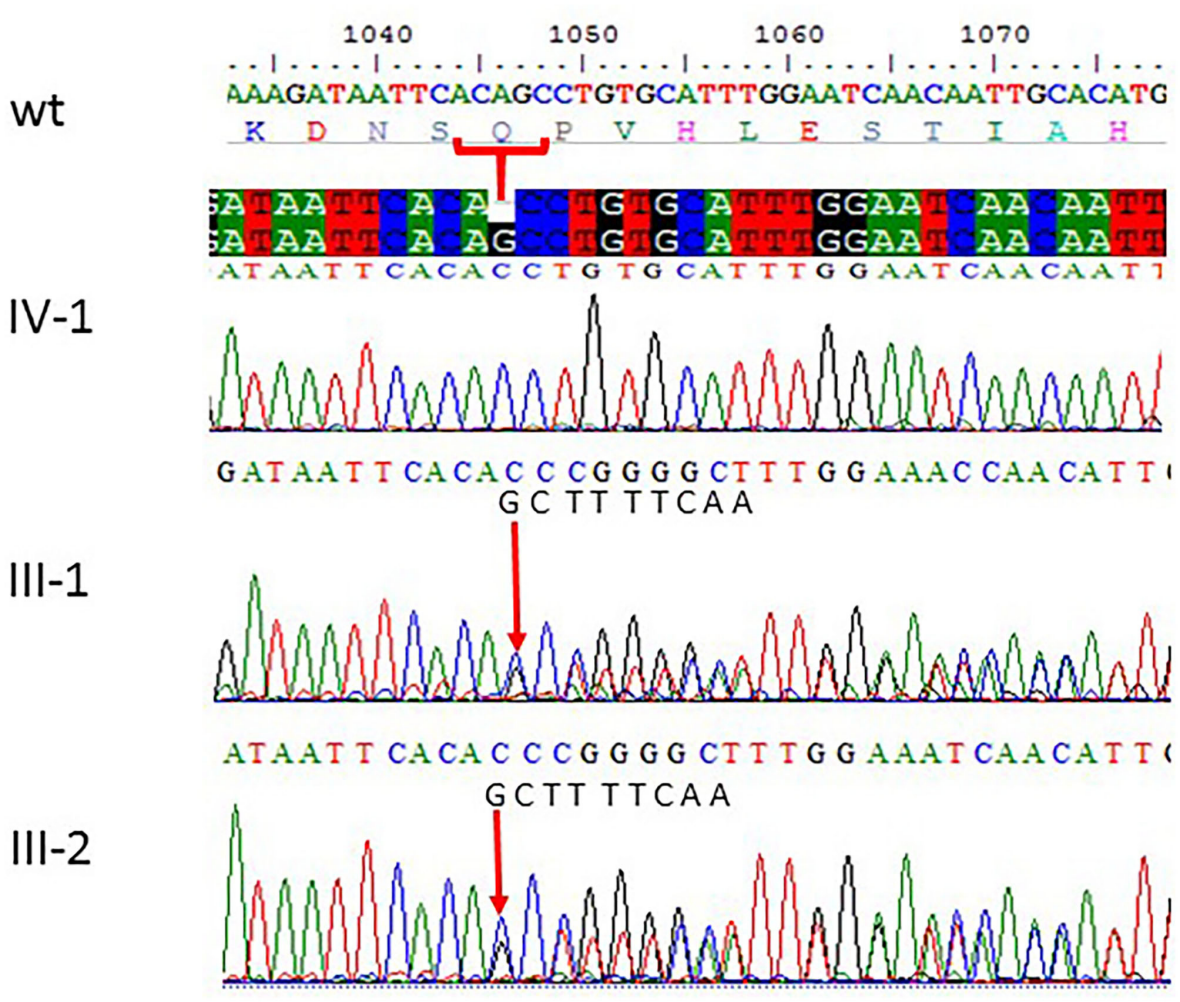
Sanger sequencing chromatograms showing c.1047del G, p.Gln349HisfsTer18 in *ASPM* gene mutation in family 2. Proband IV-1 showed deletion of “G” bp as compared with the wild-type control, while both parents III-1 and III-2 are heterozygous at this position. The wild-type normal sequence “GCTTTTCAA” was also added to show change in frameshift after mutation. The upper panel shows the sequencing of the affected member, whereas the lower panel shows the parents who are carrier individuals; the arrow is pointing at the site of mutation.

**Figure 4:**
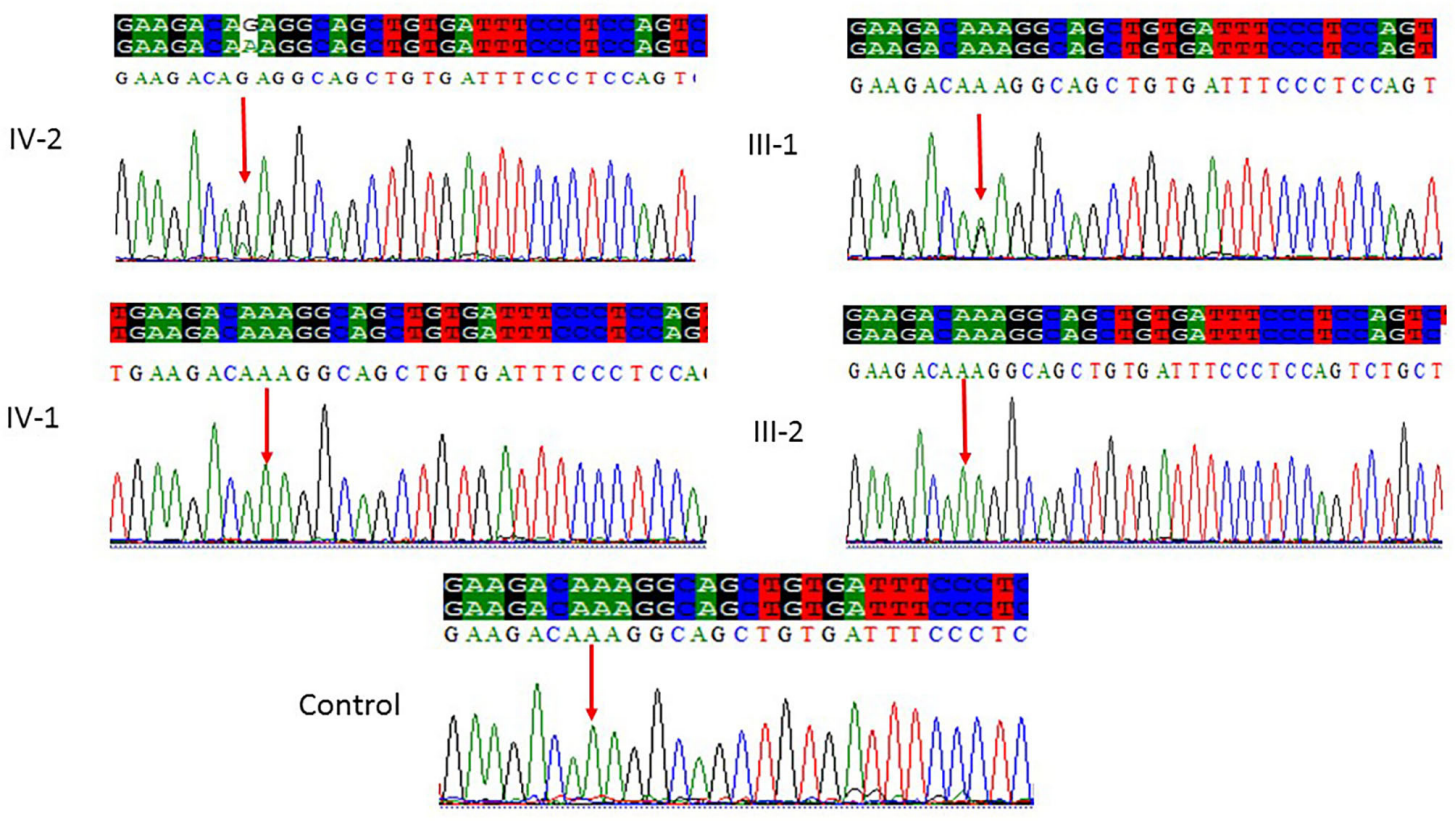
Sanger sequencing chromatograms showing a missense mutation where c.5623A>G leads to a change in protein p.Lys1875Glu in exon 18 of the *ASPM* gene in family 3. Proband IV-2 showed replacement of A>G as compared with the normal control, while one parent (III-1) is heterozygous and III-2 is homozygous, whereas the normal brother IV-1 was homozygous at this position. Panel showing the sequencing of the affected member as well as the normal brother and parents; the arrow is pointing at the site of mutation.

All the three variants obtained after WES analysis were further validated by using Sanger sequencing analysis in all the other available family members by designing the primers for the observed mutations. The Sanger sequencing results for family 1 further confirmed a homozygous frameshift mutation in the form of 1-bp deletion c.1003del. (p.Val335^*^) in exon 3 of the *ASPM* gene, while the parents were normal as shown in Figure 2. Further, the Sanger sequencing for family 2 proved c.1047del G, p.Gln349HisfsTer18 in the *ASPM* gene, while the parents were unaffected carriers as shown in Figure 3. Moreover, the Sanger sequencing for family 3 also showed similar results obtained after WES confirmed a missense mutation c.5623A>G leading to a change in protein p.Lys1875Glu in exon 18 of the *ASPM* gene as represented in Figure 4. However, both parents and the normal brother were not carriers. Finally, to find out if the identified variants are pathogenic for these families only and to rule out this possibility, Sanger sequencing for 100 unrelated control people was also done.

## Discussion

In this study, we identified three novel mutations in three different primary microcephaly families related to the *ASPM* gene from Saudi population. The identified variants perfectly segregated with the disease phenotype in each family. Previously, Bond et al. (11) identified *ASPM* mutations which are homologous to *Drosophila melanogaster* “abnormal spindle” gene (asp) and are responsible for the usual mitotic spindle functioning in embryonic neuroblast and harbor at the MCPH5 locus. Autosomal recessive variation of this gene is known to cause primary microcephaly, and the phenotype of the patient's head circumference <3 SD is related to sex and age present at birth. Microcephaly is a condition of small brain growth, and individuals with microcephaly have small brain sizes, mild to severe intellectual disability, and delayed speech and language skills, though some unusual individuals with a minor condition of microcephaly and having a normal aptitude have also been reported. Furthermore, some other clinical features may include short stature or mild seizures. Motor skills, such as standing, sitting, and walking, may also be mildly delayed. Based on the referral notes for patient involved in this study, primary microcephaly could be of relevance for the reported microcephaly.

Recent studies showed that mutations in *ASPM* at MCPH5 are common in Saudi population, which is in line with previous reports showing that the detection rate of *ASPM* mutations is 59.5% (22/37) of the determined MCPH in different populations (13, 25, 26).

*ASPM* mutations have been reported in the HGMD professional 2017.4 database with a total of 146 pathogenic variants. Most of these mutations are missense/nonsense (64 variants), small deletions (58 variants), splicing mutations (12 variants), small insertions (9 variants), gross deletions (2 variants), and complex rearrangement (1 variant), and these pathogenic variants have been reported to cause primary microcephaly, MCPH5, and microcephaly with intellectual disability. Some studies proposed that mutations of the *ASPM* result in an unstable RNA that activates nonsense-mediated RNA decay (NMD); on the other hand, other studies suggested mutations of *ASPM* synthesis truncated protein (27–29). Although the precise mechanism that underlies microcephaly caused by pathogenic *ASMP* mutations still has not been fully elucidated, both mechanisms result in a marked reduction of *ASPM* protein in neuronal cells, which results in reduced fetal brain growth and microcephaly.

In our study, three novel variants were identified: frameshift mutation c.1003del. (p.Val335^*^) in exon 3 of *ASPM*; deletion variant c.1047del G, p.Gln349HisfsTer18; and *de novo* mutation c.5623A>G leading to a change in protein p.Lys1875Glu in exon 18 of the *ASPM* gene. All these three variants detected in this study were classified under variants of unidentified clinical implication according to the latest guidelines of the ACMG (30).

Due to the clinical phenotype of the patients studied here involving microcephaly between −3 and −5 SD of head circumference and intellectual disability and following the MRI reports of a characteristic small frontal lobe, we postulated that these three variants are pathogenic and cause primary microcephaly. In this study, we reported three novel mutations in the *ASPM* gene causing primary microcephaly. Further, functional analysis of these variants is suggested and it is also suggested that WES analysis is successful in molecular diagnostic studies especially in those populations with a high rate of cousin marriages. Further, population screening of primary microcephaly families is required for better understanding and management of this disease for efficient and accurate genetic testing will be helpful for patient management and to reduce disease risk through genetic counseling in the population. In conclusion, the three novel mutations identified in our study will further enlarge the mutation dataset in ASPM-related MCPH and offer new insight into the types and frequencies of *ASPM* mutations in Saudi population, which will further help to identify innovative strategies to tackle primary microcephaly.

## Data Availability Statement

The datasets for this article are not publicly available because family consents to share data publicly was not allowed. Requests to access the datasets should be directed to Muhammad Imran Naseer, mimrannaseer@yahoo.com.

## Ethics Statement

The studies involving human participants were reviewed and approved by Center of Excellence in Genomic Medicine Research King Abdulaziz University Jeddah, Saudi Arabia. Written informed consent to participate in this study was provided by the participants' legal guardian/next of kin. Written informed consent was obtained from the individual(s), and minor(s)' legal guardian/next of kin, for the publication of any potentially identifiable images or data included in this article.

## Author Contributions

MN, SA, and AC designed the experiments and finally revised the manuscript. AA and MN conducted the experiments. OM, HA, SS, and MN analyzed the data. MN and SA wrote the manuscript. All authors contributed to the editing of the manuscript and the scientific discussions.

## Conflict of Interest

The authors declare that the research was conducted in the absence of any commercial or financial relationships that could be construed as a potential conflict of interest.
